# *Ace-1 *duplication in *Anopheles gambiae*: a challenge for malaria control

**DOI:** 10.1186/1475-2875-8-70

**Published:** 2009-04-18

**Authors:** Luc Djogbénou, Pierrick Labbé, Fabrice Chandre, Nicole Pasteur, Mylène Weill

**Affiliations:** 1 Institut de Recherche pour le Développement, UR 016, 01 BP 4414 RP Cotonou, Benin; 2 Centre de Recherche Entomologique de Cotonou (CREC), 06 BP 2604 Cotonou, Benin; 3 Institute of Evolutionary Biology, University of Edinburgh, Edinburgh, UK; 4 Université Montpellier 2 – CNRS, Institut des Sciences de l'Evolution, Equipe Génétique de l'Adaptation, C.C. 065, Place Eugène Bataillon, 34095 Montpellier, France

## Abstract

**Background:**

Insecticide resistance is a rapid and recent evolutionary phenomenon with serious economic and public health implications. In the mosquito *Anopheles gambiae s.s*., main vector of malaria, resistance to organophosphates and carbamates is mainly due to a single amino-acid substitution in acetylcholinesterase 1 (AChE1). This mutation entails a large fitness cost. However, a resistant duplicated allele of the gene encoding AChE1 (*ace-1*), potentially associated to a lower fitness cost, recently appeared in *An. gambiae*.

**Methods:**

Using molecular phenotype data collected from natural populations from West Africa, the frequency of this duplicated allele was investigated by statistical inference. This method is based on the departure from Hardy-Weinberg phenotypic frequency equilibrium caused by the presence of this new allele.

**Results:**

The duplicated allele, *Ag*-*ace-1*^*D*^, reaches a frequency up to 0.65 in Ivory Coast and Burkina Faso, and is potentially present in Benin. A previous study showed that *Ag*-*ace-1*^*D*^, present in both M and S molecular forms in different West Africa countries, was generated by a single genetic event. This single origin and its present distribution suggest that this new allele is currently spreading.

**Conclusion:**

The spread of this less costly resistance allele could represent a major threat to public health, as it may impede *An. gambiae *control strategies, and thus increases the risk of malaria outbreaks.

## Background

Since early 1950s, humans have controlled the populations of many agricultural or medical arthropod pests, mostly with chemical insecticides. After years of success, evolutionary adaptations to these new conditions began to occur and resistance spread rapidly; more than 500 species are now resistant to at least one insecticide [1]. Insecticide resistance is a rapid and recent evolutionary phenomenon, providing insight into the processes of adaptation through natural selection, but it has serious economic and public health implications. In the arms race between arthropods and humans, the mosquito *Anopheles gambiae*, the main vector of malaria, seems to have just moved up a gear with the emergence of a resistant duplicated allele of the gene encoding acetylcholinesterase 1 (AChE1).

AChE1 is a critical enzyme in nerve transmission and the target of two of the most commonly used types of insecticides (organophosphates, OPs, and carbamates, CXs). Like several other mosquito species (including *Culex pipiens*, the well-studied vector of West Nile virus), *An. gambiae *displays resistance due to a single amino-acid substitution, from a glycine to a serine at the position 119, in the AChE1 catalytic site (G119S)[2]. In *C. pipiens*, there is direct and indirect evidence that the resistance allele (*ace-1*^*R*^) entails a large fitness cost, probably due to the mutated AChE1 having a much lower level of activity. Homozygous *ace-1*^*R *^mosquitoes survive in the presence of insecticide, but are rapidly outcompeted in the absence of insecticide (see review in [3]). Heterozygotes are subject to smaller costs than resistant homozygotes in the absence of insecticide. In treated areas, they survive better than susceptible homozygotes, but are less resistant than *ace-1*^*R *^homozygotes. Due to the patchy nature of mosquito control, the generalist heterozygote is advantaged across treated and non-treated areas, although the more specialist resistant and susceptible homozygotes are locally selected in treated and non-treated environments respectively. Moreover, heterozygotes cannot invade due to the segregation burden leading to the loss of the advantage in half of their progeny.

Several duplicated alleles (*ace-1*^*D*^) have recently appeared, which link a susceptible and a resistant copy of the *ace-1 *gene on the same chromosome [4]. Duplication thus creates a "permanent heterozygote" allele. The first case of *ace-1 *gene duplication was recently discovered in *An. gambiae *[5]. Molecular analysis showed this duplicated allele (*Ag*-*ace-1*^*D*^) to be present at several sites and to have probably spread among the two molecular forms S and M of *An. gambiae s.s*, by introgression.

Unfortunately, it is not possible to design a simple test for studying the frequency of *Ag*-*ace-1*^*D *^due to the lack of features specific to this duplication, as with available genotyping methods carriers of this duplicated allele cannot be distinguished from classical heterozygotes. Thus an indirect method previously developed for *C. pipiens *was used to estimate *Ag*-*ace-1*^*D *^frequency in the field [6]. The results of this analysis and the potential consequences for *An. gambiae *population management and on malaria control are discussed.

## Methods

### Data collection

**Figure 1:**
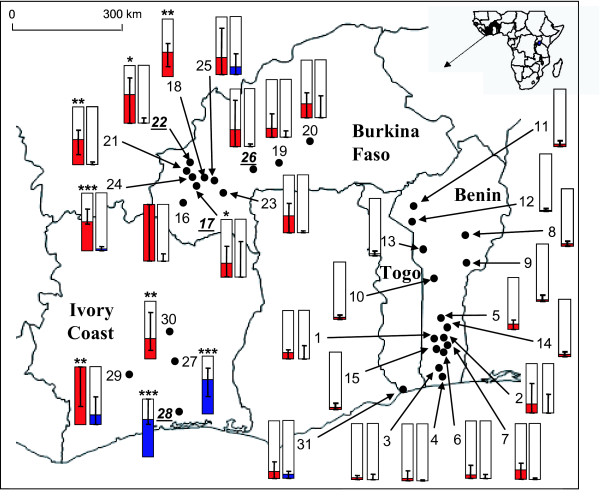
***Ag-ace-1*^*D *^frequency in Western Africa**. The frequency of *Ag-ace-1*^*D *^is given for each *An. gambiae *molecular form: M (red) and S blue). Samples are described in Table 1. Samples in which *Ag-ace-1*^*D *^was detected by molecular analysis are bolded and underlined (Table 2). Significant presence of the duplicated allele (before Bonferroni correction, see Methods) is given with * for *P *< 0.05, ** for *P *< 0.01 and *** for *P *< 0.001.

The study area is shown in Figure 1. Both published data [5,7] and data from new samples were used. The date and location of the sampling sites are shown in Table 1. For each locality, several ponds where sampled in an area a few hundred meter-squares to insure a representative sample of the local population.

**Table 1:** Sample data

**#**	**Locality**	**Country**	**Sampling date**	**Ref**
1	Abomey	Benin	june 06	[5]
2	Bohicon	Benin	may 06	[5]
3	Houegbo	Benin	apr 06	This study
4	Niaouli	Benin	apr 06	This study
5	Paouignan	Benin	june 06	[5]
6	Zogbodomey	Benin	may 06	[5]
7	Cana	Benin	may 06	This study
8	Bembereke	Benin	oct-07	This study
9	Parakou	Benin	oct-06	This study
10	Bassila	Benin	oct-07	This study
11	Tanguieta	Benin	oct-07	This study
12	Natitingou	Benin	oct-07	This study
13	Djougou	Benin	oct-07	This study
14	Dassa	Benin	oct-07	This study
15	Savalou	Benin	oct-07	This study
16	Darsalamy	Burkina Faso	aug 06	[7]
17	*Dioulassoba*	Burkina Faso	apr 06	[5,7]
18	Kuinima	Burkina Faso	apr 06	[7]
19	Mombamba	Burkina Faso	aug 06	This study
20	Sabou	Burkina Faso	aug 06	This study
21	Samandeni	Burkina Faso	aug 06	[7]
22	*Séguéré*	Burkina Faso	aug 06	[5,7]
23	Soumousso	Burkina Faso	aug 06	This study
24	Vallée du Kou	Burkina Faso	apr 05	[7]
25	Yegueresso	Burkina Faso	aug 06	[7]
26	*Boromo*	Burkina Faso	aug 06	[5]
27	Toumodi	Ivory Coast	sept-04	[5]
28	*Niamoue*	Ivory Coast	sept-04	[5]
29	Toumbokro	Ivory Coast	sept-04	[5]
30	Yaokoffikro	Ivory Coast	sept-04	[5]
31	Lomé	Togo	march 05	This study

For each population shown in Fig. 1 (the corresponding number is indicated in column #), the country, sampling date and study of reference are given.

### Molecular analysis

All samples were collected at the larval stage and reared to adulthood in the laboratory. Genomic DNA was extracted from each field mosquito. The protocol used is a simplified version of Collins *et al*. [8]: a single mosquito is homogenized in a 1.5 ml Eppendorf tube containing 200 μl of CTAB buffer (100 mM Tris HCL, pH 8.0, 10 mM EDTA, 1.4 M NaCl, 2% CTAB) and incubated at 65°C for 5 min; then 200 μl of chloroform are added. After centrifugation (room temperature, 5 min, 12000 g), the supernatant is moved to a fresh tube, 200 μl of iso-propyl alcohol are added, and the mix is centrifuged again (12000 g, 15 min). After discarding supernatant, the pellet is washed with 70% ethanol, dried and resuspended in DNAse Free water. The molecular form of each individual was determined by a PCR-based test, as described in [9]. The *ace-1 *genotype was assessed by RFLP analysis (see [5,7]).

### Statistical analyses

The presence of a duplicated allele causes an apparent excess of heterozygous [RS] phenotypes and thus a departure from the Hardy-Weinberg proportions expected with two alleles only (*ace-1*^*R *^and *ace-1*^*S*^) [6]. This departure is related to the frequency of the duplicated allele and was used to estimate *Ag*-*ace-1*^*D *^frequency in *An. gambiae *populations. The presence of *Ag*-*ace-1*^*D *^was investigated by fitting two models to the phenotypic data for each sample independently: i) a two-alleles-only model *(ace-1*^*R *^and *ace-1*^*S*^) and ii) a three-allele model, adding the duplicated allele *Ag-ace1*^*D*^. The frequency of the duplicated allele was estimated from the excess of heterozygotes observed in each sample, assuming that this excess was due exclusively to the presence of *Ag-ace-1*^*D *^[6]. This method is not as accurate as a direct identification of genotypes, but the two methods gave highly concordant results for field samples of *C. pipiens *[6]. This indirect estimate of *Ag-ace1*^*D *^frequency may be biased if the genotypes are not in Hardy-Weinberg equilibrium. However, such a bias is not expected as *An. gambiae *populations large size prevents drift and as no overdominance leading to heterozygote excess was ever found for resistance [10]. Moreover, previous studies of neutral markers in *An. gambiae *show either no departure from Hardy-Weinberg expectations or a deficit in heterozygotes, but never an excess, ensuring that this method is conservative (e.g. [11,12]).

For each sample, the expected phenotypic distributions were calculated for the S and M molecular forms, using allelic distributions and assuming the *ace-1 *locus to be at Hardy-Weinberg equilibrium. Phenotype was considered to be a three-state random variable ([RR] corresponding to (R/R) genotype, [RS] corresponding to (R/S), (D/S), (D/R) and (D/D) genotypes, and [SS] corresponding to (S/S) genotype). The log-likelihood of a sample was calculated from the phenotypic multinomial distribution. Let *n*_*ij *_and *f*_*ij *_be the observed number and expected frequency of individuals with phenotype *i *in population *j*, respectively. The log likelihood *L *of observing all the data is proportional to



*L *was maximized, using the Metropolis algorithm [13,14]. Model likelihoods were compared using *F*-tests: by construction, the three-allele model has a higher likelihood, but the presence of the duplication is considered to be confirmed only if the likelihood of this model is significantly higher than that of the two-allele model (significant *P*-value). The support limits for the frequency of each allele were also estimated. Finally, the *P*-values obtained were corrected for multiple testing, using Hommel's sequential Bonferroni correction [15]. The different samples from each country were also pooled to get a higher statistical power at a larger geographical scale (Table 2), and the same analysis as for independent collection sites has been done. As pooling data from different populations is likely to result in a heterozygote deficit (Wahlund effect), this analysis is likely to underestimate any global excess of heterozygotes [6], making the detection of such excess more significant.

**Table 2:** *Ag-ace-1*^*D*^**frequency in West Africa**.

			**M form**					**S form**			
					
**#**	**Locality**	***N***	**R**	**S**	**D**	***P*-value**	***N***	**R**	**S**	**D**	***P*-value**
1	Abomey	3	-	1	-	-	68	0	0.87	0.13	0.144 ^NS^
2	Bohicon	2	-	1	-	-	3	0	0.82	0.18	0.654 ^NS^
3	Houegbo	9	-	1	-	-	62	0	0.97	0.03	0.715 ^NS^
4	Niaouli	50	-	1	-	-	12	0	0.96	0.04	0.835 ^NS^
5	Paouignan	0	-	-	-	-	41	0	0.9	0.1	0.352 ^NS^
6	Zogbodomey	13	-	1	-	-	9	0	0.94	0.06	0.808 ^NS^
7	Cana	38	-	1	-	-	26	0	0.83	0.17	0.227 ^NS^
8	Bembereke	0	-	-	-	-	62	0	0.96	0.04	0.647 ^NS^
9	Parakou	0	-	-	-	-	20	0	0.97	0.03	0.873 ^NS^
10	Bassila	0	-	-	-	-	76	0	0.97	0.03	0.68 ^NS^
11	Tanguieta	0	-	-	-	-	47	0	0.96	0.04	0.673 ^NS^
12	Natitingou	0	-	-	-	-	48	0	0.99	0.01	0.918 ^NS^
13	Djougou	0	-	-	-	-	46	0	0.97	0.03	0.750 ^NS^
14	Dassa	0	-	-	-	-	64	0	0.96	0.04	0.652 ^NS^
15	Savalou	0	-	-	-	-	29	0	0.96	0.04	0.789 ^NS^
	**Total Bénin**	115	-	1	-	-	221	0	0.91	0.09	0.052 ^NS^
16	Darsalamy	7	-	1	-	-	2	0	0	1	0.096 ^NS^
17	*Dioulassoba*	1	-	1	-	-	23	0.21	0.55	0.24	0.044 *
18	Kuinima	0	-	-	-	-	27	0	0.58	0.42	0.002 **
19	Mombamba	8	-	1	-	-	7	0	0.85	0.15	0.563 ^NS^
20	Sabou	2	-	1	-	-	14	0	0.76	0.24	0.198 ^NS^
21	Samandeni	20	-	1	-	-	25	0	0.57	0.43	0.002 **
22	*Séguéré*	10	-	1	-	-	8	0	0.5	0.5	0.049 *
23	Soumousso	32	-	1	-	-	12	0	0.71	0.29	0.153 ^NS^
24	Vallée du Kou	86	0	0.96	0.04	0.641 ^NS^	80	0.16	0.34	0.51	0.000 ***
25	Yegueresso	8	0	0.87	0.13	0.592 ^NS^	2	0	0.71	0.29	0.560 ^NS^
26	*Boromo*	38	0.05	0.95	0	1.000 ^NS^	2	0	0.71	0.29	0.560 ^NS^
	**Total Burkina Faso**	212	0.03	0.97	0	1.000 ^NS^	202	0.12	0.53	0.35	0.000 ***
27	Toumodi	18	0	0.41	0.59	0.001 ***	0	-	-	-	-
28	*Niamoue*	24	0.35	0	0.65	0.000 ***	0	-	-	-	-
29	Toumbokro	19	0.23	0.61	0.16	0.195 ^NS^	5	0	0	1	0.008 **
30	Yaokoffikro	0	-	-	-	-	19	0.32	0.32	0.35	0.009 **
	**Total Ivory Coast**	61	0.26	0.4	0.34	0.000 ***	24	0.29	0.29	0.42	0.001 ***
31	Lomé (Togo)	73	0	0.93	0.07	0.391 ^NS^	13	0	0.88	0.12	0.531 ^NS^

For each population sampled, the number of mosquitoes of each of the S and M molecular forms of *An. gambiae *is given, together with the estimated frequency of the various alleles: R, S and D for *ace-1*^*R*^, *ace-1*^*S *^and *ace-1*^*D*^, respectively. A global estimation is also presented for each country sampled. The populations in italics are those in which *Ag-ace-1*^*D *^has been identified by molecular analysis [5]. Finally, the *p*-value for the test for the presence of *ace-1*^*D *^is also given for each population (see Methods), with ^NS ^for *P *≥ 0.05, * for *P *< 0.05, ** for *P *< 0.01 and *** for *P *< 0.001 (except when no estimate was possible, *i.e*. when all the mosquitoes of a sample were susceptible). The *P*-values that remained significant after Bonferroni correction (see methods) are presented in bold; *P*-values no longer significant after Bonferroni correction are shown in italics.

## Results and discussion

The frequency of the recently discovered duplicated allele of the *ace-1 *gene in *An. gambiae*, *Ag*-*ace-1*^*D*^, was investigated in natural populations from West Africa by considering the departure from Hardy-Weinberg proportions caused by its presence [6].

Figure 1 shows the predicted spatial distribution of *Ag*-*ace-1*^*D *^in the S and M forms of *An. gambiae*, as shown by previous molecular investigations and analyses of heterozygote excess. The probability of *Ag*-*ace-1*^*D *^being present was significant in nine samples (five after Bonferroni correction) from Ivory Coast (four samples) and Burkina Faso (five samples), in both M (two samples) and S (seven samples) molecular forms of *An. gambiae *(Figure 1 and Table 2). In these samples, the frequency of *Ag*-*ace-1*^*D *^was up to 0.65, with the lowest significant frequency being 0.24, consistent with the expected highly conservative output of the method used. Indeed, this method will detect low frequencies only in large samples; for example, *Ag-ace-1*^*D *^was not detected with this method in one of the analysed populations (Boromo, population #26, Table 2), whereas molecular methods proved this duplication to be present [5]. The frequency and the geographic distribution of this duplication are therefore probably underestimated. For example, the analysis of each Benin population independently did not provide any indication supporting the presence of the duplication in this country (Figure 1 and Table 2). Nevertheless, the pooled analysis yields a *P*-value of 0.052, which points toward the potential presence of *Ag-ace-1*^*D *^as this method underestimate the excess of heterozygotes and thus its frequency. However, more data are required to confirm the presence of the duplicated allele in Benin (Table 2). The complete lack of variation of the *Ag*-*ace-1*^*D *^sequence over several countries [5] indicates that this allele was generated by a single genetic event and its current distribution suggests that it is probably spreading.

Unfortunately, the spread of this new resistance allele poses a potential major threat to public health, as *An. gambiae *is the main vector of malaria. Indeed, several studies of a similar allele in *C. pipiens *have indicated that the duplication entails a lower fitness cost than the single-copy resistance gene, *ace-1*^*R *^[4,6] (but see [16]). This is probably also the case for *An. gambiae*, as the mutated AChE1 gene is also associated with a strong decrease in enzyme activity [17]. The presence and spread of the *Ag*-*ace-1*^*D *^allele may greatly impede *An. gambiae *control strategies designed to maintain resistance alleles at low frequencies through the use of different insecticides with no cross-resistance in a mosaic or rotation strategy. It has been clearly demonstrated [18,19] that the efficiency of such strategies increases with the fitness cost of resistance.

## Conclusion

Insecticides for controlling malaria vectors are a major weapon in the battle between humans and malaria. Unfortunately, these insecticides exert strong selection pressure on vector populations, causing the spread of resistance genes, such as the resistance allele observed at the *ace-1 *locus in *An. gambiae*. The long-term use of an insecticide promotes the selection of new resistant variants, with a high risk of selecting a low (or null)-cost variant. The *ace-1 *duplicated allele recently appeared in *An. gambiae *is probably an example of such a low-cost variant. It is shown here that the presence of this duplicated allele, known from the molecular analysis of a few mosquitoes in some samples from Burkina Faso and Ivory Coast [5] is largely distributed in several countries of Western Africa, sometimes at high frequencies, and that it is probably spreading.

To prevent such spreads of resistance genes, it is crucial to develop the largest possible number of complementary means of control (e.g. larval insecticides, mosquito nets, biological agents, etc.) and to use them wisely. However, the emergence of *ace-1 *duplication in natural populations of *An. gambiae*, has just given mosquitoes the edge in this particular battle, seriously undermining our efforts to control vector populations and increasing the risk of malaria outbreaks.

## Competing interests

The authors declare that they have no competing interests.

## Authors' contributions

LD designed the study, acquired the data and wrote the manuscript. PL analysed the data and wrote the manuscript. NP, FC and MW contributed to the design of the study and for draft and revision of the manuscript. All authors read and approved the final manuscript.

## References

[B1] Bills P (2001). A new database of pesticide resistant insects and mites (Arthropods). Pesticide notes.

[B2] Weill M, Lutfalla G, Mogensen K, Chandre F, Berthomieu A, Berticat C, Pasteur N, Philips A, Fort P, Raymond M (2003). Insecticide resistance in mosquito vectors. Nature.

[B3] Weill M, Labbé P, Duron O, Pasteur N, Fort P, Raymond M, Fellowes MDE, Holloway GJ, Rolff J (2005). Insecticide resistance in the mosquito *Culex pipiens *: towards an understanding of the evolution of *ace *genes. Insect evolutionary ecology.

[B4] Labbé P, Berthomieu A, Berticat C, Alout H, Raymond M, Lenormand T, Weill M (2007). Independent duplications of the acetylcholinesterase gene conferring insecticide resistance in the mosquito *Culex pipiens *. Mol Biol Evol.

[B5] Djogbénou L, Chandre F, Berthomieu A, Dabire R, Koffi A, Alout H, Weill M (2008). Evidence of introgression of the *ace-1 *^*R*^mutation and of the *ace-1 *duplication in West African *Anopheles gambiae *s. s. PLoS ONE.

[B6] Lenormand T, Guillemaud T, Bourguet D, Raymond M (1998). Appearance and sweep of a gene duplication: adaptive response and potential for new functions in the mosquito *Culex pipiens *. Evolution.

[B7] Djogbénou L, Dabire R, Diabate A, Kengne P, Akogbeto M, Hougard JM, Chandre F (2008). Identification and geographic distribution of the *ace-1 *^*R *^mutation in the malaria vector *Anopheles gambiae *in South-Western Burkina Faso, West Africa. Am J Trop Med Hyg.

[B8] Collins FH, Mendez MA, Rasmussen MO, Mehaffey PC, Besansky NJ, Finnerty V (1987). A ribosomal RNA gene probe differentiates member species of the *Anopheles gambiae *complex. Am J Trop Med Hyg.

[B9] Favia G, Torre Ad, Bagayoko M, Lanfrancotti A, Sagnon NF, Touré YT, Coluzzi M (1997). Molecular identification of sympatric chromosomal forms of *Anopheles gambiae *and further evidence of their reproductive isolation. Insect Mol Biol.

[B10] Djogbénou L, Weill M, Hougard JM, Raymond M, Akogbéto M, Chandre F (2007). Characterization of Insensitive Acetylcholinesterase (*ace-1 *^*R*^) in *Anopheles gambiae *(Diptera: Culicidae): Resistance Levels and Dominance. J Med Entomol.

[B11] Lanzaro GC, Touré YT, Carnahan J, Zheng L, Dolo G, Traoré S, Petrarca V, Vernick KD, Taylor CE (1998). Complexities in the genetic structure of *Anopheles gambiae *populations in west Africa as revealed by microsatellite DNA analysis. PNAS.

[B12] Lehmann T, Hawley WA, Kamau L, Fontenille D, Simard F, Collins FH (1996). Genetic differentiation of *Anopheles gambiae *populations from East and West Africa: comparison of microsatellite and allozyme loci. Heredity.

[B13] Labbé P, Lenormand T, Raymond M (2005). On the worldwide spread of an insecticide resistance gene: a role for local selection. J Evol Biol.

[B14] Lenormand T, Bourguet D, Guillemaud T, Raymond M (1999). Tracking the evolution of insecticide resistance in the mosquito *Culex pipiens *. Nature.

[B15] Hommel G (1988). A stagewise rejective multiple test procedure based on a modified Bonferroni test. Biometrika.

[B16] Labbé P, Berticat C, Berthomieu A, Unal S, Bernard C, Weill M, Lenormand T (2007). Forty years of erratic insecticide resistance evolution in the mosquito *Culex pipiens *. PLoS Genetics.

[B17] Alout H, Djogbenou L, Berticat C, Chandre F, Weill M (2008). Comparison of *Anopheles gambiae *and *Culex pipiens *acetycholinesterase 1 biochemical properties. Comp Biochem Physiol B-Biochem Mol Biol.

[B18] Carrière Y, Deland J-P, Roff DA, Vincent C (1994). Life-history cost associated with the evolution of insecticide resistance. Proc R Soc Lond B.

[B19] Lenormand T, Raymond M (1998). Resistance management: the stable zone strategy. Proc R Soc Lond B.

